# Iron Deficiency and Iron Deficiency Anemia Are Common Epidemiological Conditions in Saudi Arabia: Report of the National Epidemiological Survey

**DOI:** 10.1155/2020/6642568

**Published:** 2020-12-29

**Authors:** Tarek Owaidah, Nouf Al-Numair, Ayman Al-Suliman, Mohammed Zolaly, Rana Hasanato, Faisal Al Zahrani, Mohameed Albalawi, Layla Bashawri, Khawar Siddiqui, Faisal Alalaf, Abdulkareem Almomen, Muhammad Raihan Sajid

**Affiliations:** ^1^Department of Pathology and Laboratory Medicine, King Faisal Specialist Hospital & RC, Alfaisal University, Riyadh 11211, Saudi Arabia; ^2^Research Center, King Faisal Specialist Hospital, Alfaisal University, Riyadh 11211, Saudi Arabia; ^3^Department of Pediatric Hematology, College of Medicine, Taibah University, Medina, Saudi Arabia; ^4^Department of Pathology, College of Medicine, King Saud University, Riyadh 12372, Saudi Arabia; ^5^Department of Clinical Laboratory Sciences, College of Applied Medical Sciences, Imam Abdulrahman Bin Faisal University, Dammam, Saudi Arabia; ^6^Department of Internal Medicine, Taibah University, Medina, Saudi Arabia; ^7^Department of Pediatric Hematology/Oncology, King Faisal Specialist Hospital, Riyadh 11211, Saudi Arabia; ^8^Department of Medical Genetics, Umm Al-Qura University Faculty of Medicine, Makkah, Saudi Arabia; ^9^Department of Pathology, Alfaisal University, Riyadh 11533, Saudi Arabia

## Abstract

Iron deficiency is the most prevalent nutritional deficiency worldwide. According to an estimate by the World Health Organization, up to 27% of the world's population experience iron deficiency anemia (IDA). Studies conducted in the Middle East, including Saudi Arabia, have suggested that IDA is the most common cause of anemia, especially among females. This study aimed to determine the prevalence of IDA and iron deficiency (ID) among apparently healthy young university students from four regions in Saudi Arabia. Students were asked to complete a simple survey questionnaire; blood samples were then collected and analyzed after obtaining informed consent. A total of 981 students completed the survey, with 11% of the participants reporting symptoms of anemia; 34% of participants were diagnosed with IDA and 6% reported a diagnosis of hemoglobinopathy. Blood analysis confirmed the prevalence of ID and IDA in 28.6% and 10.7% of the participants, respectively; those with ID and IDA were mostly females (88.5% and 94%, resp.). Thalassemia trait and sickle cell trait were detected in 1.3% and 7% of participants, respectively. Our findings from a national survey among young university in Saudi Arabia indicate a high prevalence of ID and IDA.

## 1. Introduction

Iron is a vital element in human metabolism and plays a central role in erythropoiesis. It is also involved in many other intracellular processes in the body tissues [1]. Iron is necessary to maintain healthy cells, skin, hair, and nails. Iron metabolism in the body is a complex process that is regulated by hormones that balance the absorption by the cells that line the gastrointestinal tract, or pool in body compartments, storage, and excretions. The daily requirement of iron for erythrocyte production and cellular metabolism is 25 mg/day, which is met through iron absorption from the diet (1-2 mg/day), iron salvaged from erythrocyte breakdown by macrophages (20–25 mg/day), and through iron stores (total of 3–5 g in adults) [2]. Iron requirements change based on physiological changes. The adolescence phase is characterized by an accelerated rate of growth and development. Iron requirements in girls begin to increase after menarche, with 30–40 mL of blood loss during each menstruation cycle leading to a loss of 15–30 mg of iron per cycle. In boys, testosterone secretion and an increase in muscular mass require additional iron [3].

Various nonmodifiable and modifiable factors exert an influence on an individual's iron balance, ranging from sociodemographic characteristics (including the individual's age, sex, marital status, level of education, income, and ethnicity) to the amount and quality of the food and beverages they consume; iron balance is also affected by individuals' mental and physical health, the medication they consume, any underlying medical conditions, and their genetic makeup [4–6].

Iron deficiency (ID) is a state in which there is insufficient iron to maintain normal physiological functions of tissues [7]. This condition results from an imbalance between iron requirements and the quantity ingested and absorbed. ID is associated with impaired physical work capacity, cognitive function, reproductive physiology, and poor pregnancy outcomes [8]. After the exhaustion of iron, the imbalance between the supply and requirement causes a decrease in the erythropoiesis leading to low Hb synthesis and anemia. Some functional changes may occur in the absence of anemia; however, most functional deficits occur with the development of anemia [9]. Functional iron deficiency (FID) describes a condition where there is insufficient iron incorporation into erythroid precursors in the face of apparently adequate body iron stores [10].

Anemia is a condition in which the number of red blood cells (consequently their oxygen-carrying capacity) is insufficient to meet the body's physiological needs. Iron deficiency anemia (IDA) is presently the most prevalent and common type of micronutrient deficiency in developing countries that results from a long-term negative iron imbalance. The World Health Organization (WHO) has reported that approximately two billion individuals worldwide suffer from anemia, with 50% of all anemia secondary to IDA [11]. Usually, ID develops gradually and does not have clinically apparent symptoms until the anemia becomes severe [12]. The literature on anemia in adolescents and youth is scarce, with most studies focusing on women and children.

Serum ferritin has been suggested as the best test for diagnosing or excluding IDA; however, the cutoff values for the diagnosis of ID are an area of debate [13–17] with the cutoff used affecting the estimation of the real prevalence of IDA. Thus far, there is no consensus on the ferritin cutoffs used to define absolute or functional ID in the general population. Although the WHO has made some recommendations for the diagnosis of ID (based on low ferritin level) and IDA (based on low Hb plus low ferritin level), most of the reported prevalence studies have used unstandardized tools for the estimation of IDA prevalence, including studies from Saudi Arabia [13–17]. Another limitation of ferritin in the diagnosis of iron deficiency has been an acute phase reactant in conditions of inflammation and infection.

In Saudi Arabia, the overall prevalence of IDA is not well established by epidemiological surveys; however, there are many reports from single institutions and for specific age or sex populations with a reported prevalence ranging from 10 to 60% [7, 18–27]. The WHO reviewed publications on the prevalence of IDA in their country profile for Saudi Arabia and found that most of the reports were on anemia in general without any clear definition of IDA. Therefore, the present study aimed to report the prevalence of ID and IDA through a national epidemiological survey among apparently healthy young university students within four regions in Saudi Arabia.

## 2. Materials and Methods

This cross-sectional study was conducted among 981 young apparently healthy Saudi university students with high socioeconomic status identified and recruited by randomized sampling from universities within four regions in Saudi Arabia (Riyadh, Medina, Makkah, and Dammam). The study sample included 507 and 474 female and male students, respectively. This study was conducted in two parts: the first part involved the administration of a questionnaire to evaluate the knowledge about anemia among the participants and to correlate these with the laboratory findings (Table 1), while the second part of the study involved the collection of blood samples for the evaluation of anemia status, to confirm the presence of ID, and to evaluate the presence of hemoglobinopathy. Some of the blood samples had to be discarded because of the poor quality of blood samples; the final laboratory analysis was performed on 956 samples.

### 2.1. Subjects

After multicenter Institutional Review Board approval, an epidemiological survey was carried out on a randomly selected sample of young adult Saudi university students of both sexes from four regions of Saudi Arabia (Riyadh, Medina, Makkah, and Dammam) between May 2016 and 2018. The participants were asked to complete a simple questionnaire. The survey was conducted on site by trained Arabic-speaking interviewers after explaining the aims of the study. After obtaining verbal consent, blood samples were collected from all the participating students. All questionnaires were coded for data entry. The study was approved by the research advisory committee of King Faisal Specialist Hospital as part of the national survey, with a science and technology grant from King Abdulaziz City.

### 2.2. Blood Sample Analysis

Blood samples were collected by trained nurses from each participant in two anticoagulants (5 mL each): EDTA and sodium heparin. Complete blood count (CBC), plasma ferritin level, and capillary zone electrophoresis were performed for each participant. CBC was estimated from the EDTA samples using an automated SYSMEX XN-10 instrument (Sysmex Corporation, Kobe, Japan). Plasma ferritin level was measured using an automated chemistry analyzer COBAS 601 (Roche Diagnostics, Basel, Switzerland), while Hb variants were detected from fresh hemolysate blood samples using capillary zone electrophoresis.

Based on the WHO criteria for the diagnosis of anemia, participants were categorized into normal, ID, and IDA groups. Normal levels were defined as Hb ≥ 12.0 g/dl for females and ≥13.0 g/dl for males, along with plasma ferritin levels ≥30.0 ng/ml. ID was defined as Hb > 12.0 g/dl for females and >13.0 g/dl for males, with a plasma ferritin level of either <15 ng/ml or <30.0 ng/ml. IDA was defined as Hb < 12.0 g/dl for females and <13.0 g/dl for males, with a plasma ferritin level <30.0 ng/ml.

### 2.3. Data Management and Quality Assurance

All participants were interviewed by Arabic-speaking trained individuals and the data were collected using specially designed Arabic-language Case Report Forms. Confidentiality was maintained by assigning each participant a unique identification number which was entered into a computerized database. Data were validated for data entry errors by cross-checking the improbable answers. Discrepancies were handled by reviewing the original forms. All data were analyzed using IBM SPSS Statistics Version 20 (IBM Corp., Armonk, NY, USA) after data cleaning and quality checks.

### 2.4. Statistical Analyses

Continuous data are presented as medians and accompanying ranges. For continuous data that did not conform with normality assumptions, an independent-sample Mann–Whitney *U* test was used to test for the significance of the difference between the two groups. For categorical data, the chi-square test or Fisher's exact test was used to test for independence of the association. The level of significance was set at 5%.

## 3. Results

Between January 2016 and June 2018, a total of 981 college students from four different cities in Saudi Arabia (representing four regions) were surveyed. There were 507 females and 474 males in our sample, with a median age of 19.5 (17.3–25.8) years for females and 18.9 (16.3–38.9) years for males. In response to the questionnaire, 11.1% (*n* = 109) of participants (73 females) indicated that they knew about their anemia status and reported different types of anemia, with IDA (33.9%) being the most common (Table 1).

**Figure 1:**
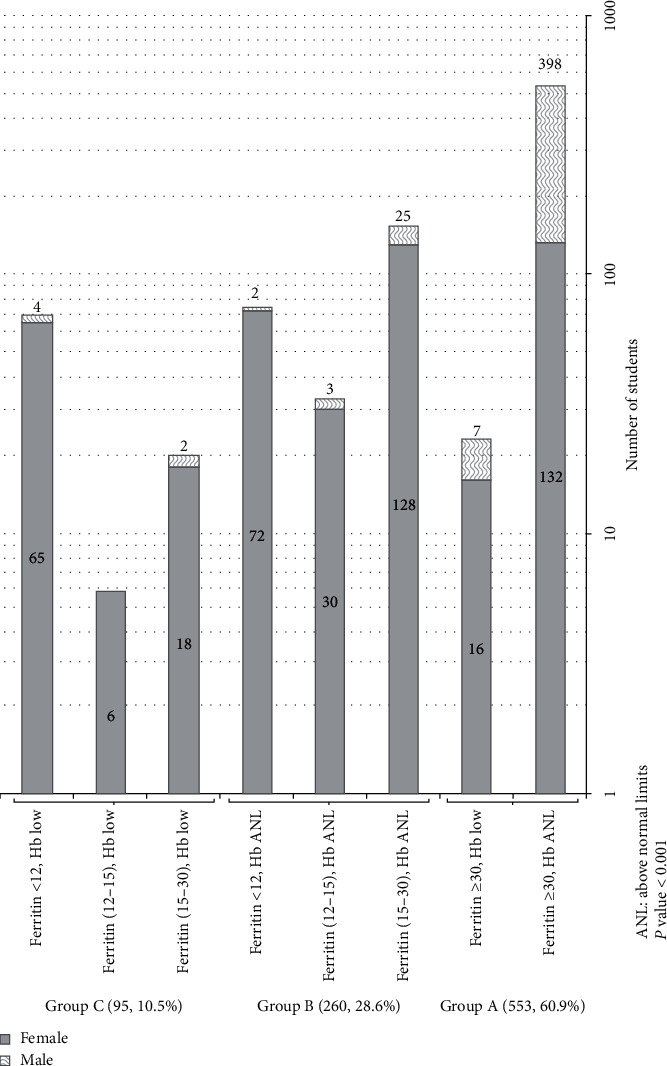
Sufficiency profile with respect to ferritin and Hb by gender.

A significant difference (*p* ≤ 0.001) in the levels of Hb, ferritin, hematocrit (HCT), platelets, mean platelet volume, Hb-A, and Hb-A2 was observed between male and female students (Table 2). The results of ferritin and Hb are presented as three groups: Groups A, B, and C (Table 3). The groups were based on three different cutoff values for ferritin; the internationally accepted cutoff value for ferritin is <30 ng/ml [15]. Additionally, we evaluated two other cutoff values based on previous reports: <15 ng/ml and <12 ng/m [12–14]. Based on these cutoffs, we found that 61.0% of students had normal Hb for sex and adequate iron stores while 5.6% of students showed anemia due to hemoglobinopathy. Different prevalence of ID and IDA was found based on the different cutoffs (Figure 1). The overall prevalence for ID and IDA was 28.1% and 10.7% with a female predominance of 88.9% and 94.1% in the two groups, respectively (Table 3).

We found that 98.5% of females and 99% of males were within the normal range (<3.5%) of Hb-A2 (normal hemoglobin type), while 1.5% of females and 1% of males had elevated values (>3.5%) of Hb-A2; the diagnosis of *β*-thalassemia trait Hb-A2 was found to be significantly different for students without ID or IDA and with ID and IDA (*p* < 0.001). There were 40 (4.1%) students with hemoglobin-S >23%, while three students had hemoglobin-S ≤23%, which could be due to coinheritance of the alpha thalassemia or another Hb variant that we could not confirm.

### 3.1. Regional Results

Regional variations in the prevalence of hemoglobinopathy, ID, and IDA were observed (Table 4). There were variations in the prevalence of hemoglobinopathy by region, with the Dammam region showing the highest prevalence (59.2%), followed by Makkah (22.4%), Riyadh (12.2%), and Medina (6.1%) (*p* < 0.001). However, we did not find any significant association between hemoglobinopathies and the incidence of ID or IDA in the overall cohort.

## 4. Discussion

Iron deficiency anemia is a major health problem worldwide, especially in developing countries [28, 29]. This study establishes the prevalence of ID and IDA among young university students from four major regions of Saudi Arabia. Serum ferritin levels decrease during the early stages of ID as iron stores are depleted, leading to uncomplicated ID. In Saudi Arabia, there are many reports about the magnitude of this national health problem; however, these reports were either from a single institute, or for specific population groups, conducted among either males or females, or were from one region. The overall reported prevalence of IDA in Saudi Arabia ranges from as low as 10% to as high as 60% [7, 18–27]. This is a unique study as it is the first large scale study to determine the prevalence of IDA in healthy young university students from Saudi Arabia. To the best of our knowledge, based on a search of PubMed and Google scholar databases, this is the first study to be conducted in four regions of Saudi Arabia simultaneously.

There are limited data on the definition of ID, with available data recommending a ferritin cutoff of <15 ng/mL with a normal hemoglobin (Hb) level for age and sex for the diagnosis of ID; this criterion seems to be specific but not sensitive [13, 14]. Pfeiffer and Looker suggested a cutoff of <12 ng/mL and observed that it was sensitive but not indicative of the severity of ID [15]. As these thresholds have not been universally adopted, the WHO has defined ID as serum or plasma ferritin levels <12 ng/mL in children younger than 5 years and less than 30 ng/mL when inflammation is concurrent. For children older than 5 years, ID is diagnosed when ferritin concentrations are <15 ng/mL [16]. Another suggested threshold that has not yet been validated but has been adopted by the Royal College of Pathologists of Australasia is a ferritin cutoff of 30 ng/mL [17].

Our results show a high frequency of ID (28.6%) among apparently healthy students. These findings are interesting and show the similarity between the central (Riyadh), eastern (Dammam), northern (Medina), and western (Makkah) regions of the country. Similar findings have been reported previously by Sinclair et al. in aerobics-trained males and females, where 33% of the participants had ID with a predominance of females (88%) [30]; similar results were also observed in our study. In a large retrospective study in the general population using ferritin level below 30 ng/mL as the cutoff to diagnose ID, Abuaisha et al. reported an ID prevalence of 57.5% and 7.6% among females and males, respectively, with overt IDA developing in 14% of the females within 5 years of follow-up [31]. Similar results have been reported in Japan, where 36–45% of women aged 20–29 years and 44–49% of women aged 30–49 years were diagnosed with ID using ferritin levels <15 ng/ml as the cutoff [32]. In the UK, 15.5% of women aged 19–64 years were found to have ID (ferritin <15 ng/mL) [33]. A study from the US National Health and Nutrition Examination Survey reported that 10.9% of women aged 18–49 years had ferritin levels <12 ng/mL [34].

The cutoff value for identifying people with ID has been the focus of many studies that looked at the impact of this deficiency on human well-being and the development of other symptoms. Although the percentage of individuals with ID who will develop IDA is not well established, Abuaisha et al. have shown in a follow-up of over 5 years that the development of IDA in females (14%) was much more than in males (0.5%) [31]. Soppi reviewed the symptoms of people with ID and reported many possible symptoms related to it, with some patients showing profound symptoms for many years before the development of IDA [35]. This study reports an overall prevalence of 10.5% for IDA as compared with previously published studies (10–60%) [7, 18–27]. The difference in methodologies used in these studies may be responsible for the observed variation. Some of these studies have used crude definitions of IDA by using MCV or Hb levels without proper estimation of iron status in the studied populations. Abalkhail and Shawky, in a facility-based study on 2,850 school children, reported the prevalence of anemia as 20.5% using Hb < 115 as the cutoff. This study did not differentiate between IDA and other types of anemias including hemoglobinopathy [7]. Two other studies based only on Hb levels reported the prevalence of anemia among teenagers in Najran (the south-western region of Saudi Arabia) (22.5% with a mean Hb value of 94 g/L) [19] and in Medina university students (with an overall prevalence of mild anemia (<110/dL) of 45%) [20]. In a small study from the western region of Saudi Arabia, Gari studied 123 children less than 12 years of age using <105 g/L and <10 *µ*g/L as the cutoffs for Hb and ferritin, respectively. He reported similar results to our study, with an observed prevalence of 25.2% and 10.6% for ID and IDA, respectively [21]. In another study in female college students from the eastern province of Saudi Arabia, Al Jamea et al. evaluated anemia in 201 students using the WHO definition of Hb and ferritin (<120 g/L and <15 *µ*g/L, resp.). They reported a prevalence of 8.2% and 28.8% for ID and IDA, respectively [36]. Alswailem et al. reported the prevalence of IDA as 41.6% in a large cohort of nonpregnant females in Riyadh City, which is much higher than our finding of 10.9%. However, this prevalence is similar to our questionnaire results, where 33.9% of the students indicated that they had IDA. This may have been because of the use of a questionnaire to report the prevalence of IDA without laboratory confirmation [37]. A study on the regional variations in the prevalence of ID and IDA indicated that the highest prevalence of ID was in the Makkah region (42.4%), while the lowest was in Medina (19.8%); for IDA, the highest prevalence was seen in Dammam (11.5%), while the lowest was in Makkah (9.8%). This variation has also been noticed in previous reports where Gari reported a prevalence of 25.2% in the Makkah region [21] while Taha et al. reported low ferritin level in 32.3% of the participants in the eastern province [38]; these results are not far from our report of 24.1%. Similar variability, though to a lesser extent, was found in IDA. Alquaiz et al. reported IDA in 49% of children (6-to-24-month-old) in the Medina region [25]. Further, 1.5% of the female students and 1% of the male students were diagnosed with *β*-thalassemia trait in the present study, which agrees with a recently reported study by Alsaeed et al. (1.3%) [39]. Additionally, the prevalence of sickle cell hemoglobin was 4.1% in our study population which is close to that reported by a study from the premarital program (2.7%) [40].

## 5. Conclusions

In conclusion, our results confirm a high prevalence of ID in all the studied regions of Saudi Arabia. IDA is a prevalent health problem in apparently healthy young adults from universities in the four regions of Saudi Arabia, with similar prevalence seen across different regions. We believe that there is an urgent need to develop effective strategies to alleviate iron deficiency and IDA in this population. Although Saudi Arabia is a wealthy country with a high socioeconomic status, there is a high prevalence of both ID and IDA. One of the limitations of this study is the lack of markers for inflammation or infection. More national surveys are required to explore the possible causes and to design preventive measures.

## Figures and Tables

**Table 1:** Responses to the survey questionnaire (*n* = 981).

Inquiry	Female (507) *n* (%)	Male (474) *n* (%)	Total (981) *n* (%)	*p* value
*Do you suffer from any type of anemia?*				
Yes	73 (14.4%)	36 (7.6%)	109 (11.1%)	0.001
G6PD	1 (20%)	4 (80%)	5 (4.6%)	
Iron deficiency/IDA	33 (89.2%)	4 (10.8%)	37 (33.9%)	
Anemia	8 (88.9%)	1 (11.1%)	9 (8.3%)	
Sickle cell	2 (50.0%)	2 (50.0%)	4 (3.7%)	
Sickle cell trait	-	2 (100%)	2 (1.8%)	
Thalassemia	1 (100%)	-	1 (0.9%)	
Spherocytosis	1 (100%)	-	1 (0.9%)	
Type unknown	27 (54.0%)	23 (46.0%)	50 (45.9%)	

*Do any of your family members have thalassemia or sickle cell disease?*				
Yes	49 (9.7%)	72 (15.2%)	121 (12.3%)	0.009

*Do you have history of parental consanguinity?*				
Yes	128 (25.2%)	171 (36.1%)	299 (30.5%)	0.1

*Have you ever been diagnosed with any bleeding disorders in the past?*				
Yes	37 (7.3%)	31 (6.5%)	68 (6.9%)	0.706
Hemophilia	2 (100.0%)	-	2 (2.9%)	
Platelet deficiency	1 (100.0%)	-	1 (1.5%)	
Don't exactly know the diagnosis	7 (46.7%)	8 (53.3%)	15 (22.1%)	

**Table 2:** Age and hematological profile of the students at the interview.

Variable	*n*	Female (*n* = 507)		*n*	Male (*n* = 474)		*p* value
		Median	(Min–Max)		Median	(Min–Max)	
Age (years)	257	19.5	(17.3–25.8)	301	18.9	(16.3–38.9)	0.349
Hb (g/dL)	501	12.6	(6.8–17.0)	468	15.1	(8.7–17.7)	0.001
Ferritin (ng/mL)	502	20.5	(1.6–393.0)	466	90.0	(4.6–543.0)	0.001
HCT (%)	500	40.0	(4.3–56.3)	468	46.0	(29.8–57.4)	0.001
MCV (fL)	501	86.0	(9.4–108.5)	468	85.2	(10.0–111.8)	0.555
Plts (10^9^/L)	490	236.0	(104–592)	455	230.0	(102–501)	0.001
MPV (fL)	499	10.0	(7.1–14.9)	467	9.3	(6.6–14.2)	0.001
HbA (%)	455	97.5	(55.5–98.7)	401	97.3	(53.9–99.2)	0.001
HbA2 (%)	457	2.5	(0.7–5.7)	402	2.6	(0.8–5.5)	0.001
HbF (%)	66	0.7	(0.2–28.9)	26	0.7	(0.1–7.1)	0.751
HbS (%)	25	35.6	(16.6–71.7)	18	34.8	(16.7–91.3)	0.941

Hb: hemoglobin; Hct: hematocrit; MCV : mean corpuscular volume; Plts: platelets; MPV : mean platelet volume; HbA: hemoglobin A; HbF: fetal hemoglobin; and HbS : an abnormal type of hemoglobin inherited from parents. None of the data conformed to the normality assumption.

**Table 3:** Observations based on hematological profile—full Cohort, all regions.

Observations	Female	Male	Total	*p* value
*For confirmed lab values (n* *=* *956)*				
Group A (ferritin ≥30)	161 (27.5%)	424 (72.5%)	585 (61.2%)	<0.001
Ferritin ≥30, Hb above normal limits^*∗*^	138	416		
Ferritin ≥30, Hb low^*∗∗*^	23	8		
Group B (low ferritin, normal Hb)	239 (88.8%)	30 (11.2%)	269 (28.1%)	
Ferritin ≥15 and <30, Hb above normal limits	136	25		
Ferritin ≥12 and <15, Hb above normal limits	30	3		
Ferritin <12, Hb above normal limits	73	2		
Group C (low ferritin and low Hb)	96 (94.1%)	6 (5.9%)	102 (10.7%)	
Ferritin ≥15 and <30, Hb low	20	2		
Ferritin ≥12 and <15, Hb low	7	0		
Ferritin <12, Hb low	69	4		

*Thalassemia*				
Hb-A2 ≤ 3.5%	450 (98.5%)	398 (99%)	848 (98.7%)	
Hb-A2 > 3.5%	7 (1.5%)	4 (1%)	11 (1.3%)	

*Sickle cell anemia*				
Hb-S ≤ 23%	2 (8%)	1 (5.6%)	3 (7%)	
Hb-S > 23%	23 (92%)	17 (94.4%)	40 (93%)	

Two students were positive for both thalassemia and sickle cell anemia. ,^*∗*^Male ≥13, female ≥12;^*∗∗*^male < 13, female <12.

**Table 4:** Observations based on the hematological profile (excluding cases of thalassemia and sickle cell disease) by regions with confirmed lab values (*n* = 908).

Regions	Group B (low ferritin, normal Hb)	Female	Male	Total	*p* value	Group C (low ferritin and low Hb)	Female	Male	Total	*p* value
Dammam	Region total	34 (81.0%)	8 (19.0%)	42/174 (24.1%)	<0.001	Region total	17 (85%)	3 (15%)	20/174 (11.5%)	0.001
	Ferritin ≥15 and <30, Hb ANL	19	5			Ferritin ≥15 and <30, Hb low	4	1		
	Ferritin ≥12 and <15, Hb ANL	5	1			Ferritin ≥12 and <15, Hb low	2	0		
	Ferritin <12, Hb ANL	10	2			Ferritin <12, Hb low	11	2		

Makkah	Region total	112 (95.7%)	5 (4.3%)	117/276 (42.4%)	<0.001	Region total	27 (100%)	0 (0%)	27/276 (9.8%)	-
	Ferritin ≥15 and <30, Hb ANL	58	4			Ferritin ≥15 and <30, Hb low	5	0		
	Ferritin ≥12 and <15, Hb ANL	20	1			Ferritin ≥12 and <15, Hb low	1	0		
	Ferritin <12, Hb ANL	34	0			Ferritin <12, Hb low	21	0		

Madina	Region total	47 (81%)	11 (19.0%)	58/293 (21%)	<0.001	Region total	29 (96.7%)	1 (3.3%)	30/293 (10.2%)	0.033
	Ferritin ≥15 and <30, Hb ANL	29	11			Ferritin ≥15 and <30, Hb low	4	1		
	Ferritin ≥12 and <15, Hb ANL	2	0			Ferritin ≥12 and <15, Hb low	0	0		
	Ferritin <12, Hb ANL	16	0			Ferritin <12, Hb low	25	0		

Riyadh	Region total	37 (86.0%)	6 (14.0%)	43/165 (26.1%)	<0.001	Region total	16 (88.9%)	2 (11.1%)	18/165 (10.9%)	0.007
	Ferritin ≥15 and <30, Hb ANL	22	5			Ferritin ≥15 and <30, Hb low	5	0		
	Ferritin ≥12 and <15, Hb ANL	3	1			Ferritin ≥12 and <15, Hb low	3	0		
	Ferritin <12, Hb ANL	12	0			Ferritin <12, Hb low	8	2		

ANL: above normal limits.

## Data Availability

The data used to support the findings of this paper are upon request to the corresponding author.
